# First Described Case of Infective Endocarditis Involving the Pulmonary Valve Caused by *Moraxella catarrhalis*

**DOI:** 10.1155/crdi/1815282

**Published:** 2025-10-15

**Authors:** P. Karamani, E. Christopoulou, A. Negash, C. Charalambous, G. M. Georgiou, I. Tzanavaros, G. Miltiadous

**Affiliations:** ^1^Department of Internal Medicine, Apollonion Private Hospital, Strovolos, Nicosia, Cyprus; ^2^Department of Cardiology, Apollonion Private Hospital, Strovolos, Nicosia, Cyprus; ^3^Cardiac Innovation Center, Apollonion Private Hospital, Strovolos, Nicosia, Cyprus

**Keywords:** *Moraxella catarrhalis*, multimodality imaging, pulmonary abscess, pulmonary valve replacement, right-sided endocarditis

## Abstract

**Background:**

*Moraxella catarrhalis*, once considered a component of the normal human flora of the upper respiratory tract, is now recognized as a true pathogen and, rarely, a cause of infective endocarditis (IE).

**Case Presentation:**

We describe a rare case of a 36-year-old woman who presented with respiratory symptoms and was initially misdiagnosed with pneumonia and *M. catarrhalis* bacteremia. No vegetations were seen on transesophageal echocardiogram (TOE) imaging. However, further investigation with cardiac computed tomography (CT) revealed prosthetic pulmonary valve IE caused by *M. catarrhalis*, which also resulted in pulmonary abscesses.

**Conclusions:**

This case highlights the importance of considering *M. catarrhalis* as a true pathogen in invasive disease, including prosthetic pulmonary valve IE. It also demonstrates the limitations of negative TOE findings in the evaluation of right-sided IE, particularly when prosthetic valves are involved. Cardiac CT is crucial when TOE results are negative but clinical suspicion for prosthetic right-sided IE remains high, as it aids in diagnosing both the infection and its perivalvular or periprosthetic complications.

**Summary**

• *Moraxella catarrhalis* can cause prosthetic pulmonary valve infective endocarditis (IE).
• Pulmonary valve endocarditis is rare and may present atypically with recurrent respiratory symptoms.
• TOE has limitations in detecting prosthetic pulmonary valve endocarditis and its complications.
• Cardiac computed tomography (CT) is an essential complementary diagnostic tool in these cases.

## 1. Introduction

*Moraxella catarrhalis* is a Gram-negativediplococcus belonging to the family Moraxellaceae. It is an exclusively human commensal and mucosal pathogen. Colonization of infants by *M. catarrhalis* is common, with rates approaching 100%, and is associated with otitis media. In contrast, only up to 5% of adults are colonized, though colonization rates are higher among individuals with chronic obstructive pulmonary disease (COPD) or other underlying respiratory conditions.

Clinically, *M. catarrhalis* most commonly causes lower respiratory tract infections in patients with COPD, pneumonia in older adults, sinusitis, and, more rarely, bacteremia, osteomyelitis, and infective endocarditis (IE) [1, 2].

A systematic review by Ioannou et al. described 31 reported cases of IE caused by Moraxella species, comprising 25 case reports and 2 case series. The mitral valve was the most commonly affected site. A prosthetic cardiac valve was present in 25.8% (8 of 31) of the patients [3]. Since that review, two additional cases have been published [4, 5]. Notably, no previous cases of pulmonary valve IE caused by Moraxella species have been reported.

Pulmonary valve IE is rare, accounting for only 1.5%–2% of all IE cases, with isolated pulmonary valve involvement being even less common. Risk factors include intravenous drug use, congenital heart disease, healthcare-associated invasive procedures, and, occasionally, immunosuppression [6].

Here, we report the first case of prosthetic pulmonary valve IE caused by *M. catarrhalis*, presenting primarily with recurrent respiratory infections due to septic pulmonary emboli.

## 2. Case Report

A 36-year-old woman, a former smoker, presented to the emergency department with fever, chills, and a productive cough that had persisted for three months. She had a history of Tetralogy of Fallot and had undergone pulmonary valve and root pulmonary artery replacement twice—first in infancy and again 5 years prior.

Following her most recent surgery, she experienced recurrent episodes of fever and chills over 4 years, sometimes self-resolving and sometimes requiring antibiotic therapy.

One month before admission, she had been hospitalized with right lower lobe pneumonia and *M. catarrhalis* bacteremia. She was treated with intravenous cefuroxime for 20 days, resulting in normalization of inflammatory markers. A transesophageal echocardiogram (TOE) performed at that time revealed no vegetations or perivalvular abscesses.

**Figure 1:**
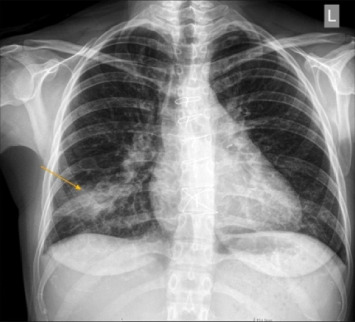
Chest x-ray revealed consolidation of right lower lobe (yellow arrow).

Upon the current presentation, her vital signs were as follows: temperature 38.6°C, heart rate 115 bpm, blood pressure 136/82 mmHg, respiratory rate 22 breaths/min, and oxygen saturation 96% on room air. Cardiovascular examination revealed a systolic murmur over the pulmonary valve area. Crackles were auscultated in the right lower lung zone. Chest x-ray showed right lower lobe consolidation (Figure 1).

Laboratory tests demonstrated the following:

- Microcytic hypochromic anemia: hemoglobin 9.8 g/dL, mean corpuscular volume 72 fL, and mean corpuscular hemoglobin 23 pg/cell
- Elevated white blood cell count: 13,430 cells/mm^3^
- Elevated C-reactive protein: 209 mg/L
- Elevated erythrocyte sedimentation rate (ESR): 48 mm/hr
- Elevated rheumatoid factor: 279 U/mL

**Figure 2:**
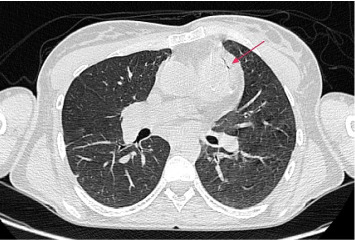
Cardiac CT scan shows fluid collection surrounded by a thick layer of inflammatory-enhancing tissue as perivalvular abscess (red arrow).

**Figure 3:**
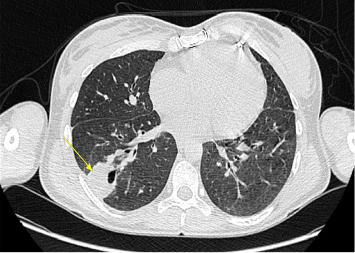
CT findings in keeping with the plain film appearances, demonstrating a cavity within the right lower lobe with the luminal surface irregular relatively extensive adjacent consolidation (yellow arrow).

Given the high suspicion of IE, a repeat TOE was performed but again showed no vegetations or other abnormalities. Subsequent cardiac computed tomography (CT) revealed a perivalvular abscess (Figure 2), while chest CT identified two pulmonary abscesses consistent with septic emboli (Figure 3).

Blood cultures obtained on admission later grew Gram-negative diplococci, identified as *M. catarrhalis* using Matrix-Assisted Laser Desorption/Ionization Time-of-Flight (MALDI-TOF) testing.

The diagnosis of prosthetic valve IE was confirmed using the 2023 modified Duke criteria based on the following [7]:

• One major criterion: imaging evidence of IE (perivalvular abscess on cardiac CT)
• Four minor criteria: predisposing condition (prosthetic pulmonary valve), fever > 38°C, pulmonary abscesses, positive rheumatoid factor, and positive blood cultures that do not meet as a major criterion.

**Figure 4:**
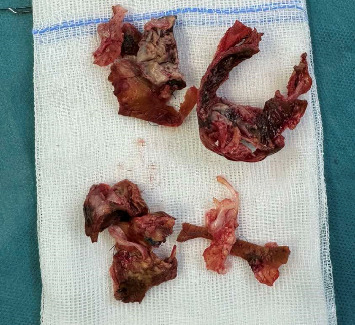
Debris of the excised homograft of pulmonary valve and root pulmonary artery.

The patient was started on intravenous meropenem and gentamycin. After 2 weeks of therapy, she underwent repeat pulmonary valve and root pulmonary artery replacement (Figure 4). Cultures of excised valve tissue were negative.

A follow-up chest CT showed significant improvement in the pulmonary abscesses. Based on microbiological findings, moxifloxacin was continued until normalization of inflammatory markers and resolution of radiographic abnormalities.

## 3. Discussion

This is the first reported case of prosthetic pulmonary valve IE caused by *M. catarrhalis*. The patient's recurrent episodes of pneumonia were, in retrospect, caused by septic pulmonary emboli originating from right-sided IE. Septic emboli are a common complication of right-sided IE, occurring in up to 82% of the cases [8]. As a result, Bamford et al. suggested that clinicians should consider chest CT routinely as part of right-sided IE follow-up [9]. Indeed, repeated CT chest in our patient had showed resolution of lung abscesses in full accordance with the normalization of the inflammatory blood parameters.

Beta-lactam antibiotics are known as first-line empirical therapy for *M. Catarrhalis* infections, including IE [10]. In our patient, initial treatment with beta and third-generation cephalosporins likely controlled the pneumonia episodes but was insufficient for eradication of IE, as treatment durations were inadequate for endocarditis. This explains the recurrence of septic emboli and persistent pulmonary complications. The cultures of excised valve tissue were negative likely due to prior 2 weeks of successful antibiotic treatment.

Echocardiography is the first-line imaging modality for suspected IE. TOE is recommended when transthoracic echocardiogram (TTE) findings are inconclusive, especially in patients with prosthetic valves [11]. However, TOE is less effective for evaluating the pulmonary valve due to its anatomical location and, in case of prosthetic valve, due to acoustic shadowing caused by prosthetic components [12].

Cardiac CT has proven superior to TOE in detecting perivalvular and periprosthetic complications such as abscesses, pseudoaneurysms, and fistulas. In one study of 115 patients with surgically confirmed IE, TOE missed 52% of perivalvular abscesses [13]. Cardiac CT and MRI are also increasingly used for detailed anatomical evaluation of pulmonary valves and surrounding structures [11].

In this case, the negative TOE results delayed diagnosis, whereas cardiac CT provided the definitive evidence needed to guide diagnosis, treatment, and surgical intervention.

## 4. Conclusions

This case underscores several critical points as follows:

- *Moraxella catarrhalis* should be recognized as a true pathogen capable of causing invasive disease, including prosthetic pulmonary valve IE.
- Right-sided IE can present atypically with recurrent respiratory symptoms such as pneumonia.
- When TOE is negative but clinical suspicion remains high, cardiac CT should be performed to evaluate for prosthetic pulmonary valve involvement and associated complications.
